# A battery of self-screening instruments and self-reported body frame could not detect eating disorders among college students

**DOI:** 10.1186/s13104-019-4672-7

**Published:** 2019-09-23

**Authors:** Norika Hayakawa, Satoshi Tanaka, Naoko Hirata, Sachiko Ogino, Norio Ozaki

**Affiliations:** 1 Nanzan University, Nagoya, 466-0824 Japan; 2 Department of Psychiatry, Nagoya University Hospital, Nagoya, 466-8560 Japan; 3 Toyoake-shi Government Office, Toyoake, 470-1112 Japan; 4 Aisei Century Hospital, Nagoya, 457-8515 Japan; 5 Nagoya University Graduate School of Medicine, Nagoya, 466-8550 Japan

**Keywords:** Eating disorder, Questionnaire, Prevalence, College students, Eating Attitudes Test

## Abstract

**Objective:**

Although studies have shown inconsistent results in terms of prevalence of eating disorders, the Eating Attitudes Test (EAT-26) was used to screen students for abnormal eating behaviors. The results of the self-reported EAT-26 and body frame, as well as the efficacy of using self-administered questionnaires (SAQs) were examined to detect eating disorders in new college students.

**Results:**

An anonymous questionnaire (EAT-26) was provided to 7738 new students; 4552 (58.8%) responders were included in the final analysis. Semi-structured interviews were conducted for 131 (1.7%) students. Among them, 6 students showed a high EAT-26 score, but were not diagnosed with an eating disorder based on the Structured Clinical Interview for DSM-IV Axis I Disorders (SCID-I). Three students were diagnosed with an eating disorder using SCID-I, but their EAT-26 scores were below the threshold. From these results, in a non-clinical population, findings on EAT-26 do not agree with those on SCID-I in terms of the diagnosis of eating disorders, and this battery is not appropriate for detecting eating disorders.

## Introduction

Community-based prevalence studies indicate that the number of people having sub-clinical eating disorders [1] is much larger than those actually diagnosed with it. Prevention, early detection, and early therapeutic intervention [2–5] are important because once an eating disorder develops, patients often lack the motivation to recover or may resist therapy and support.

Among the psychometric tools and batteries developed to detect abnormal eating behaviors [6–9], the Eating Attitudes Test 26 (EAT-26) is a low-cost and precise screening tool used worldwide. It was originally developed by Garner and Garnkel as a 40-item questionnaire for assessing clinical symptoms of eating disorders [10, 11]. Even its Japanese version [12] is consistent in terms of reliability and external validity.

Although several studies in Japan have used EAT-26 to detect eating disorders and abnormal eating behaviors at the peak age of onset among female students (i.e., high school and college students) [13–16], the results on its prevalence have been inconsistent owing to the small sample sizes and the single use of self-administered questionnaires (SAQs). Several reported findings outside Japan using both the EAT-26 and structured interviews to precisely determine the prevalence of these disorders also yielded uncertain and inconsistent results; for example, Rauof et al. did not test its external validity directly [17]. Rivas et al. reported that the EAT-26 had good specificity but insufficient sensitivity to detect eating disorders [18].

First, can questionnaire-based studies detect eating disorders precisely in the general population? To examine if the EAT-26 along with a semi-structured interview could detect eating disorders at the peak age of onset among Japanese college students, we performed a prevalence study with a relatively large sample size.

## Main text

### Methods

Additional file 1 shows the flow of the study. All new students were recruited to participate in this study from a single college in Japan having two separate campuses. They were recruited in 2012 on one campus, and from 2013 to 2015 on both campuses. The purpose of the study, its methods, and how data from questionnaires would be used were communicated in writing to all the participants. While most participants completed the questionnaires anonymously; those who agreed to undergo a semi-structured interview had to provide their names and contact information. After the questionnaires were retrieved, the staff contacted each participant who had consented to an interview, and explained verbally and in writing, the purpose, methods and how data would be used.

#### Anonymous questionnaire survey

EAT-26 is a SAQ that reveals abnormal eating behaviors. It consists of 26 items with six components scored from 0 to 3 (Zero: “Never,” “Rarely,” and “Sometimes”; 1: “Often”; 2: “Very often”; and 3: “Always”). The total score ranged from 0 to 78, and a score ≥ 20 was considered to represent abnormal eating attitudes or behaviors [10, 11].

The EAT-26 questionnaire was distributed to 7738 new college students from 2012 to 2015, during their college entrance medical checkup. Students who agreed to participate in the study, had to complete the questionnaire and place it in a collection box in a sealed envelope, while those who did not consent were asked to place blank forms.

Respondents (n = 5275, 68.2%) had to provide self-reported body weight and height on the questionnaire. BMI was calculated from these data and classified by the standards of the World Health Organization for people ≥ 20 years [19].

#### Semi-structured interview

We contacted 131 subjects (1.7%) who agreed to undergo a semi-structured interview. A clinical psychologist or a psychiatrist conducted the interviews that consisted of questions on SCID-I, module H, that covered the diagnosis of eating disorders. All interviews took place in a private room.

#### Statistical analysis

Participating students’ demographics were compared using their t-test scores. Comparisons were made between males and females, in general as well as those with eating disorders based on EAT-26 and SCID-I findings, respectively. BMIs of participants with high (≥ 20) versus low EAT-26 scores were compared using a Mann–Whitney U test. All data were analyzed with Excel 2013 (Microsoft Corp., Redmond, WA, USA) and JMP 12.0 for Macintosh (SAS Institute Japan, Tokyo, Japan). In these analyses, p < 0.05 was considered statistically significant.

### Results

A total of 5275 (68.2%) students completed the questionnaire. Forms with missing values on any item of EAT-26 (n = 241), sex (n = 43), and body weight or height (n = 473) were excluded from the data analysis. Table 1 shows participants’ demographics. The age and BMI of males were significantly higher than females, while EAT-26 scores were significantly higher in females than in males. EAT-26 scores of 40 males (2.2%) and 122 females (4.4%) were ≥ 20 (Additional file 2). Among these participants, male students were significantly older and had a significantly higher BMI and significantly lower Eating Disorder Inventory (EDI)-26 score than female students.

**Table 1:** Demographics of the study participants (n = 4552) who answered the questionnaire

	Males (n = 1800)	Females (n = 2752)	p value (Student’s t test)
Age (years)			
Mean ± SD	18.2 ± 0.7	18.1 ± 0.4	< 0.0001
Median (range)	18.0 (17.0–27.0)	18.0 (17.0–29.0)	–
BMI (kg/m^2^)			
Mean ± SD	21.1 ± 2.8	20.1 ± 2.1	< 0.0001
Median (range)	20.7 (13.3–43.1)	19.8 (9.3–33.3)	–
EAT-26 score			
Mean ± SD	3.7 ± 5.4	5.8 ± 6.8	< 0.0001
Median (range)	2.0 (0–78.0)	4.0 (0–56.0)	–

The 825 students (18.1%) classified as underweight (BMI < 18.5), included 246 males (13.7%) and 579 females (21.0%), while the 218 students (4.8%) classified as overweight (BMI ≥ 25.0), included 139 males (7.7%) and 79 females (2.9%). The majority of the students (3509; 77.1%) were classified as normal weight (BMI ≥ 18.5 to < 25).

Among the 162 students with high EAT-26 scores (40 males and 122 females), the break-up was as follows: underweight: 1 male (2.5%) and 12 females (9.8%); overweight: 12 males (30.0%) and 6 females (4.9%); and normal weight: 27 males (67.5%) and 104 females (85.2%).

Additionally, the median BMI (22.4 kg/m^2^) of male students with high EAT-26 scores was significantly higher (p < 0.0001) than that of male students with low EAT-26 scores (20.7 kg/m^2^). The same trend was seen in female students (median BMI 20.6 kg/m^2^ versus 18.6 kg/m^2^, respectively; p < 0.0001).

A total of 131 students underwent a semi-structured interview. Their demographics are shown in Table 2. Six female students had high EAT-26 scores (≥ 20), but did not meet the criteria for eating disorders as described by SCID-I (Table 3).

**Table 2:** Demographics of participants who underwent a semi-structured interview

	Males (n = 4)	Females (n = 127)
Age (years)		
Mean ± SD	18.0 ± 0.0	18.2 ± 0.8
Median (range)	18.0 (18.0–18.0)	18.0 (18.0–26.0)
BMI (kg/m^2^)		
Mean ± SD	24.0 ± 2.3	20.4 ± 2.1
Median (range)	23.8 (22.0–26.4)	20.0 (15.8–28.5)
EAT-26 score		
Mean ± SD	4.8 ± 8.2	6.3 ± 6.6
Median (range)	1.0 (0–17.0)	5.0 (0–32.0)

**Table 3:** Profiles of students with a high EAT-26 score (≥ 20) and a lack of diagnosis on SCID-I (n = 6)

	EAT-26	Sex	BMI (kg/m^2^)	SCID-I eating disorder diagnosis
Student 1	28	Female	19.5	No
Student 2	32	Female	23.4	No
Student 3	22	Female	23.9	No
Student 4	27	Female	18.0	No
Student 5	25	Female	22.0	No
Student 6	30	Female	20.3	No

*SCID-I* Structured Clinical Interview for DSM-IV Axis I

Only two students diagnosed with an eating disorder by SCID-I showed a low EAT-26 score. Of them, the one was diagnosed with bulimia nervosa (BN), but was suspected of having a history of diagnostic migration from AN. She had a normal BMI and an extremely low EAT-26 score. The other was also diagnosed with BN with normal BMI and had an EAT-26 score below the cut-off point.

### Discussion

#### Anonymous EAT-26 survey

Females had significantly higher EAT-26 scores than males, which is consistent with previous reports [20–23]. This may mean that female students in this generation are keenly interested in dieting and thus tend to experience abnormal eating attitudes and behaviors.

A high EAT-26 score was observed in 2.2% of the males and 4.4% of the females (3.6% of the total). Previous studies in Japan have reported various rates of high (≥ 20) EAT-26 scores. Mase et al. reported high EAT-26 scores in 3.2% of female college and university students [22], Makino et al. reported high EAT-26 scores in 5.1% of female college students [23], and Sasai et al. reported high EAT-26 scores in 8.7% of female college students [14]. Okamoto et al. reported that 0.7–0.9% of male students and 1.9–2.0% of female students showed high EAT-26 scores from 2002 to 2010 [24]. These results from Japanese students show a lower prevalence of high EAT-26 scores than results of similar age students outside Japan, in which scores ranged from about 15 to 17% [7, 9]. These inconsistencies may be due to differences in ethnicity (our study included mostly Asian islanders), cultural differences, or subtle differences in age or survey methods (e.g., questionnaires sent by post or hand-delivered, anonymous or non-anonymous). In addition, results may have differed if other studies included educational documents for eating disorders with the EAT-26. Constarelli and Patsai reported that EAT-26 scores tend to get higher during the examination period in colleges compared to a control period [25]. Results of our study may have been biased because it was done at the time of entrance to college, which can result in low levels of stress after passing the admission exam.

In this study, participants classified as underweight had low EAT-26 scores, potentially suggesting that underweight students may not declare abnormal eating attitudes or behaviors. A similar potential bias based on a different subjective survey was reported by Beglin and Fairburn [26], and a previous study hypothesized that these biases could be related to psychological denial or hesitancy in reporting abnormal eating behaviors in non-anonymous questionnaires [24].

#### Semi-structured interview

Only four male participants underwent a semi-structured interview; therefore, the findings from our semi-structured interview survey are limited actually to female students. The prevalence of a high EAT-26 score in the semi-structured interview was 4.6% (n = 6), that was close to the results of the anonymous questionnaire. Five out of these six students’ BMI was in the normal range. No student having a high EAT-26 score was diagnosed as having an eating disorder based on SCID-I. However, 2 females (1.5%) whose BMI was in normal range were diagnosed with an eating disorder based on a low EAT-26 score. Using SCID-I, EAT-26, and Bulimic Inventory Test, Edinburgh (BITE) for Japanese college students (n = 357), Hisamatsu et al. reported that the sensitivity of EAT-26 was low (51.2%), while the combination of EAT-26 and BITE improved the sensitivity to 80.5% [27]. Jacobi et al. reported that almost all screening tools for abnormal eating behaviors are suitable for clinical cases, but that these tools are not appropriate for the identification of at-risk eating behaviors [28].

Our findings as well as those from previous studies reveal that when a battery of SAQs like EAT-26, self-reported BMI, and semi-structured interviews are used in a non-clinical population, it is not possible to identify subjects with an eating disorder. Better screening tools and objective physical measurements of height and weight are needed to provide a more accurate diagnosis.

## Limitations

- Analysis were limited to only participants who had agreed to participate in the survey; hence, the prevalence of disease is likely to be underestimated.
- SAQs and self-declared body frame are dependent on participants’ honesty, hence in principle, their accuracy is limited.
- Only 1.7% of new students were included in our semi-structured interview, so the results could not be statistically analyzed and hence cannot be generalized to the entire population.

## Supplementary information

**Additional file 1.** Study flow and participants.

**Additional file 2.** Numbers of students from questionnaire survey, classified by EAT-26 results, gender and BMI range. Low EAT-26 score: <20, high EAT-26: ≥20.

## Data Availability

The original dataset of this article cannot be shared based on the decision of the ethics review committee.

## Abbreviations

EAT-26: Eating Attitudes Test
SAQs: self-administered questionnaires
SCID-I: Structured Clinical Interview for DSM-IV Axis I Disorders
BMI: body mass index
EDI: Eating Disorder Inventory
BITE: Bulimic Inventory Test, Edinburgh
AN: anorexia nervosa
BN: bulimia nervosa
