# Supplementary figures and images for: A battery of self-screening instruments and self-reported body frame could not detect eating disorders among college students
Source: BMC Res Notes. 2019 Sep 23;12:613. doi: 10.1186/s13104-019-4672-7 (PMC6757401; doi:10.1186/s13104-019-4672-7)

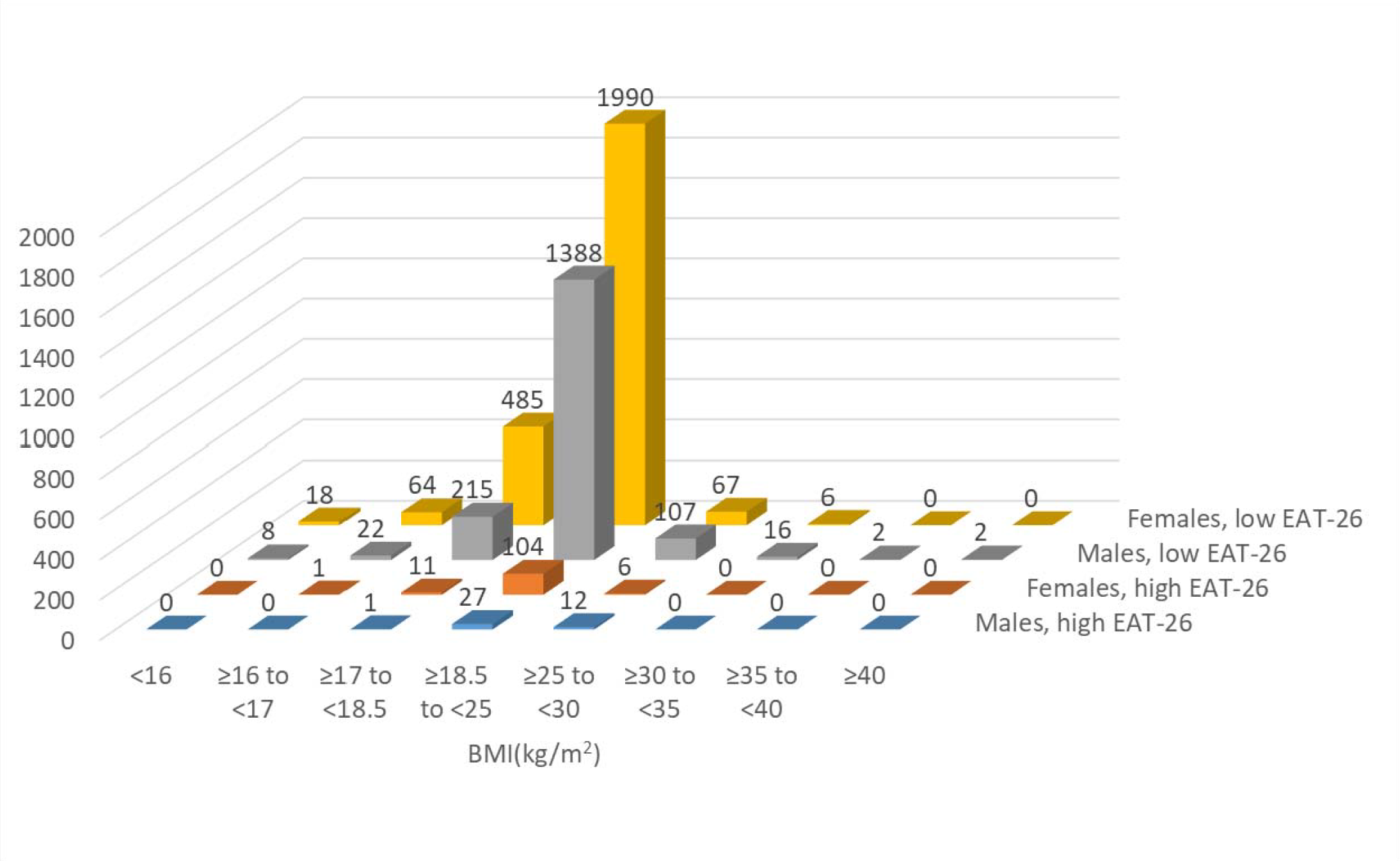

Supplement: Supplementary file 2 — Additional file 2. Numbers of students from questionnaire survey, classified by EAT-26 results, gender and BMI range. Low EAT-26 score: <20, high EAT-26: ≥20. [file 13104_2019_4672_MOESM2_ESM.tiff]
